# Estimating the Burden of Disability From Road Traffic Injuries in 5 Low- and Middle-Income Countries: Protocol for a Prospective Observational Study

**DOI:** 10.2196/40985

**Published:** 2023-02-01

**Authors:** Mohammad K Khalaf, Heather E Rosen, Sudeshna Mitra, Kazuyuki Neki, Leah Watetu Mbugua, Adnan A Hyder, Nino Paichadze

**Affiliations:** 1 Department of Global Health Milken Institute School of Public Health The George Washington University Washington, DC United States; 2 Global Road Safety Facility World Bank Washington, DC United States

**Keywords:** road traffic injuries, road traffic crashes, trauma care, disabilities, rehabilitation environmental factors, low- and middle-income countries

## Abstract

**Background:**

Road traffic injuries (RTIs) are a leading cause of death and unintentional injuries globally. They claim 1.35 million lives and produce up to 50 million injuries each year, causing a major drain on health systems. Despite this high burden, there is a lack of robust data on the long-term consequences of RTIs, specifically the level of disability experienced by many survivors and its impact on their everyday lives.

**Objective:**

This study aims to characterize RTIs, disability level, and related consequences affecting adult road traffic crash survivors in 5 low- and middle-income countries (LMICs). In addition, this study estimates the role of demographic and crash- and treatment-related factors in predicting adverse outcomes and disability as well as examining the disability level among patients with RTIs, likelihood of return to normal life, and the environmental factors that may influence these outcomes after discharge from the hospital.

**Methods:**

This prospective observational study was conducted at selected hospitals in Bangladesh, Cambodia, Ethiopia, Mexico, and Zambia. The study sample included all adult patients with RTIs admitted to the hospital for at least 24 hours. Consecutive sampling was performed until the minimum required sample size of 400 was reached for each participating country. Data were collected from patients or their caregivers using a hospital-based surveillance tool administered at the participating sites as well as a telephone-based follow-up instrument administered 1, 3, and 6 months after discharge. Descriptive analysis and multivariate models will be used to estimate the contribution of a range of factors in predicting adverse outcomes, disability, and return to normal life.

**Results:**

Enrollment began in June 2021 and was completed in April 2022. Follow-up data collection ended in September 2022. Data analysis is currently underway, with results expected for publication in mid-2023. Expected results include estimates of disability among patients with RTIs as well as identifying the predictors of adverse outcomes, disability, and the likelihood of return to normal life.

**Conclusions:**

Research findings will help better understand the long-term burden of disability from RTIs in the 5 LMICs and the challenges facing survivors of road traffic crashes. They will be used to inform interventions aimed at improving the health care, social, physical, and policy conditions in LMICs that can facilitate recovery and rehabilitation for patients with RTIs, reduce the burden of disability, and enhance their participation in society.

**International Registered Report Identifier (IRRID):**

DERR1-10.2196/40985

## Introduction

### Background

Road traffic crashes are a leading cause of trauma admissions in hospitals worldwide [1-3]. They claim 1.35 million lives and cause up to 50 million injuries each year, causing a major burden on health systems and other services [4]. According to the Global Burden of Disease Study, between 1990 and 2019, road traffic injuries (RTIs) were among the top 10 contributors to the increase in global disability adjusted life years (DALYs) [5]. In 2019, RTIs ranked seventh among the leading causes of global DALYs for all age groups and first for people aged 10 to 49 years [5].

Despite some recent improvements in global road safety, there have been persistent disparities in the burden of RTIs across world regions [4,6]. In 2019, RTIs accounted for 3.8% of the total deaths in Africa, 3.0% in the Eastern Mediterranean, 2.4% in South-East Asia, 2.3% in the Western Pacific, 2.2% in the Americas, and 0.8% in Europe [6]. In addition, RTI-induced death and disability are disproportionately higher in low- and middle-income countries (LMICs) than in high-income countries [6]. As in many other aspects of health, disparities exist in the risks and consequences affecting people with RTIs. For example, RTIs are the leading cause of death among children and young adults aged between 5 and 29 years [7]. They disproportionately affect males aged <29 years, who are far more likely to be involved in road crashes compared with females, and people from lower socioeconomic backgrounds have a higher likelihood of RTI-induced death and injury [7]. Other risk factors include driving speed; vulnerability of road users (eg, pedestrians and motorcyclists); nonuse of safety equipment (ie, motorcyclist helmets, seatbelts, and child seats); distracted driving; driving under the influence of alcohol or drugs; unsafe road infrastructure; unsafe vehicles; inadequate enforcement of traffic laws; and inadequate postcrash care [7]. These risk factors not only increase the likelihood of a road traffic crash but are also linked to the characteristics and impact of RTIs themselves. This research landscape on road traffic crashes in LMICs demonstrates important associations between crash circumstances and the consequences of RTIs. Studies have shown that crash circumstances (eg, method of transportation to the hospital, type of road user, alcohol use, and type of vehicle) are associated with morbidity and mortality after an RTI [8-13].

Research findings on RTI-induced disabilities demonstrate a high prevalence of disability among patients with RTIs in LMICs and highlight marked disparities in the experiences of injury, disability, and socioeconomic impact linked to sociodemographic, crash, and injury characteristics of patients with RTIs [10,12,14-18]. Despite their burden, many LMICs lack reliable and high-quality surveillance data on RTIs [4,19]. Only 66% of middle-income countries and 74% of low-income countries have trauma registry systems [20]. Surveillance data on the rate and severity of RTI-induced disabilities are also lacking in LMICs [4]. In addition, differences in methodologies and approaches to disability measurement underscore the continued need for more data to understand the consequences of RTIs beyond initial clinical outcomes [10,12,21,22]. The World Health Organization International Classification of Functioning, Disability, and Health framework defines disability as the interaction between a health condition (ie, an injury, disease, or disorder) and contextual factors, which include both personal and environmental factors. According to the International Classification of Functioning, Disability, and Health, environmental factors have a direct effect on disability outcomes caused initially by health conditions [23]. However, little is known about the impact of environmental barriers on the functioning of patients with RTIs after the initial treatment. Thus, a thorough understanding of disability after receiving trauma care is necessary to understand the extent to which environmental factors, such as access to medical and rehabilitation services, social support, and the physical environment, contribute to the recovery of patients with RTIs and the severity of their disability.

### Goal of This Study

The primary goal of this study is to develop a clear understanding of RTIs and their consequences in LMICs to inform advocacy actions and policies. The specific aims of this study are to describe the characteristics and initial treatment outcomes of RTIs; estimate the role of demographic, crash-related, and treatment-related factors in predicting injury severity and disability-related outcomes; and estimate the impact of environmental barriers on the level of disability experienced by patients with RTIs after hospital discharge. Our study focuses on measuring constructs within disability (activities and participation) as well as the environmental barriers that may affect them to generate the evidence needed to inform interventions and policies that address the specific needs of patients with RTIs after treatment.

## Methods

### Study Design and Settings

This prospective observational study was conducted at 9 selected hospitals in Bangladesh, Cambodia, Ethiopia, Mexico, and Zambia (Table 1). According to the Global Burden of Disease report, the 2019 death rate (per 100,000) due to RTIs was 5.41 in Bangladesh, 17.53 in Cambodia, 8.56 in Ethiopia, 16.89 in Mexico, and 12.53 in Zambia [24]. By contrast, the number of DALYs per 100,000 lost due to nonfatal injuries was 549.61 in Bangladesh, 1006.57 in Cambodia, 546.35 in Ethiopia, 945.19 in Mexico, and 768.33 in Zambia [24].

**Table 1:** Selected countries and cities.

World Bank region, country, name of hospital				City		City population^a^, n
**East Asia and Pacific**						
	**Cambodia**					
		Calmette Hospital; Preah Kossamak Hospital	Phnom Penh		1,952,000	
**Latin American and Caribbean**						
	**Mexico**					
		General Hospital of Axochiapan “Dr. Ángel Ventura Neri”	Axochiapan, Morelos		39,174	
		General Hospital of Cuautla “Dr. Mauro Belaunzarán Tapia”	Cuautla, Morelos		187,118	
		General Hospital of Cuernavaca “José G. Parres”	Cuernavaca, Morelos		1,043,000	
		General Hospital of Temixco “Enf. María de la Luz Delgado Morales”	Temixco, Morelos		122,263	
**South Asia**						
	**Bangladesh**					
		National Institute of Traumatology and Orthopedic Rehabilitation	Dhaka		19,578,000	
**Sub-Saharan Africa**						
	**Ethiopia**					
		Addis Ababa Burn Emergency and Trauma Hospital	Addis Ababa		4,400,000	
	**Zambia**					
		University Teaching Hospital	Lusaka		2,524,000	

^a^On the basis of 2018 United Nations, Department of Economic and Social Affairs population estimates for metropolitan areas (Dhaka and Cuernavaca), urban agglomerations (Phnom Penh and Lusaka), and cities proper (Addis Ababa and Kyiv) [25]. Axochiapan, Cuautla, and Temixco (Mexico) populations are based on 2020 estimates from the National Institute of Statistics and Geography (INEGI) [26].

Countries were selected to include representation in each region of the world where the World Bank operates, although we were unable to include a site from the Middle East and North Africa. Ukraine was originally selected as the site for Europe and Central Asia; however, we could not include it in the study because of the current conflict affecting the country. Hospitals were identified and selected based on previous research collaborations between national research teams and the study authors. Where possible, hospitals that were designated as trauma centers or that were present in large urban centers were prioritized. Some countries have included >1 hospital to achieve the desired sample size.

### Eligibility Criteria

The study population included adults (aged ≥18 years) of either sex admitted to the hospital because of a moderate to severe RTI, which is defined as the injury being severe enough to require hospitalization for at least 24 hours. Study participants included patients admitted to the emergency department as well as other relevant hospital departments or units (eg, neurosurgical or orthopedic surgery units). Participants were able to give consent or had a suitable proxy who gave consent on their behalf if they were unable to do so. Individuals were excluded from the study if they were aged <18 years, were discharged <24 hours after being admitted to the hospital, or were unable to communicate verbally and did not have a proxy.

### Study Sample

For the hospital surveillance portion of the study, we estimated the proportion of moderate to severe injuries in the target population of patients with RTIs. The required sample size for each country was calculated using the following equation:

*n* = *p* × (1 – *p*) × (*z* / *e*)^2^
**(1)**

where *n* is the sample size required for a large population, *p* is the proportion of the population sustaining severe injuries from a road traffic crash, *z* is the confidence level, and *e* is the margin of error. *p* is based on the estimate that 30% of the adult study population may sustain moderate to severe injuries from road traffic crashes according to studies in LMICs [10,12,21]. *z* is defined as 1.96 (for a 95% confidence level [α]) and *e* at 5%. With these considerations, the sample size is calculated to be 323, which indicates that this is an adequate sample size for estimating the proportion of moderate to severe injuries from the total population of patients with RTIs in participating hospitals. Given the sample size calculations and accounting for 20% of the combined refusal and attrition, a minimum target sample size of 400 was set for each participating country. Consecutive sampling was done such that every patient who met the inclusion criteria was selected until the required sample size was reached at the participating hospital or hospitals in each country.

For the disability portion of the study that follows the recruited patients with RTI at 3 time points within 6 months after discharge, the sample included those patients who were identified at the first follow-up. Using α value of 0.05, a sample of 320 (based on 20% loss to follow-up 6 months after discharge), and a 0.5 baseline measure adjustment (correlation of repeated measures), the study has 99% statistical power to detect a 0.3 SD difference in the mean disability score between patients without a supportive environment (ie, those who report facing environmental barriers) compared with those with a supportive environment.

### Recruitment and Data Collection

#### Overview

Patients were screened for inclusion and exclusion criteria by the data collectors at each facility at the following two points: (1) upon arrival at the emergency department and (2) at discharge from any department of the hospital. This allowed us to capture patients who came directly to the hospital, as well as patients who were transferred from other facilities and bypassed the emergency department.

Two instruments that were used for data collection are a hospital-based RTI surveillance tool and a follow-up disability-related assessment questionnaire (Table 2). The hospital-based RTI surveillance tool includes the following: (1) general patient information, (2) prehospital care, (3) RTI details, (4) initial clinical assessment and care provided, (5) disposition, (6) payment information, and (7) disability history.

**Table 2:** Data collection instruments.

Instrument	Content	Data source	Data collection method	Data collection time points
Hospital-based RTI^a^ surveillance tool	Sociodemographic characteristics, crash circumstances, prehospital care provided, clinical assessment, and disposition	Medical records	In-person	During hospital stay
Hospital-based RTI surveillance tool	Cost or payment information and history of disability	Patient or proxy interview	In-person	During hospital stay (day of discharge)
Disability assessment and environmental factors tool	Demographic characteristics, activity limitations, participation restrictions, use of or need for assistive devices, perceived return to preinjury activities, and environmental factors	Patient or proxy interview	Phone-based	1, 3, and 6 months after discharge

^a^RTI: road traffic injury.

The disability follow-up questionnaire consists of 3 modules. Module A includes selected questions from the short form of the World Health Organization Disability Assessment Survey 2.0 (WHODAS 2.0), which is a general disability instrument that measures limitations in performance in 6 life domains that are understanding and communicating, getting around, self-care, getting along with people, life activities, and participation in society [27]. Module B includes questions pertaining to returning to normal life and work and questions about assistive devices. Module C, which was administered only at 3- and 6-month follow-up time points, includes selected questions from the short form of the Craig Hospital Inventory of Environmental Factors (CHIEF) version 3 [28]. CHIEF measures perceived physical, social, and political barriers to societal participation for people with disabilities [28].

Informed consent was obtained from the patients (or proxy) in the hospital for enrollment in the study. Both study instruments were pretested with 5 to 7 patients in each participating country before the start of data collection.

#### Hospital-Based Data Collection

As part of RTI hospital surveillance, data was collected by obtaining information from the patient’s medical record and by conducting interviews with the patient (or caregiver) directly. We aimed to extract the following information from the medical record, depending on the availability (Textbox 1): sociodemographic information (age, sex, education, employment status, current partnership status, total number of persons living in household, and annual household income); prehospital care (if care was provided, by whom, type of care provided, mode of arrival at hospital, and transport time to hospital); RTI details (type of road, type of vehicle, mobile use, counterpart, and safety equipment use); and initial clinical assessment and care provided (vital signs, initial Glasgow Coma Scale, suspected alcohol and drug use, treatment, region of injury, pathology, operation, and number of days in the intensive care unit). We collected the following information through patient (or proxy) interviews: payment information (if a fee was paid, amount paid, type of service paid for, hospitalization cost, means of payment, and annual household income) and disability history (if the patient had a disability before RTI, severity of the disability, and domains affected by the disability). We also attempted to gather data from the patient or proxy if it was missing in the medical records, as appropriate.

**Variables from hospital-based surveillance tool.**

**Sociodemographic variables**

- Age group in years (18-24, 25-44, 45-64, 65-74, and >75)
- Sex (male or female)
- Educational level (no formal education, primary, secondary, college or university, professional, graduate, vocational or technical, and other)
- Employment status (daily-wage laborer, salary worker, self-employed, military, homemaker, student, retired, beggar, unemployed, unable to work, and other)
- Partnership or marital status (currently married, cohabiting, separated, divorced, widowed, and never married)
- Number of persons in household
- Annual household income (local currency)

**Prehospital care variables**

- Care provided at scene (yes, no, or unknown)
- Who provided care (person involved, bystander, relative, friend, police, ambulance staff, emergency medical technician, unknown, and other)
- What care was provided (C-spine immobilization, fracture immobilization, control of bleeding, wound care, Cardiopulmonary Resuscitation, intravenous fluids, and other)
- Mode of arrival to hospital (walk-in, auto-rickshaw, car, ambulance, taxi, motorized 2-wheeler, and other)
- Transport time to hospital (0-30 min, 30 min-1 hour, 1-2 hours, 2-6 hours, 6-24 hours, and >24 hours)

**Crash characteristics (road traffic injury [RTI] details)**

- Time of injury (day, evening, and night)
- Type of road (highway, main road, side street, village road, and other)
- Type of road user (pedestrian, driver, passenger, and other)
- Type of vehicle (car, minibus or van, bus, bicycle, motorcycle, truck or lorry, auto-rickshaw, and other)
- Mechanism of injury of pedestrian (boarding or exiting bus, boarding or exiting other vehicle, standing or walking on side of road, and crossing the road)
- Counterpart (car, minibus or van, bus, animal, auto-rickshaw, motorcycle, bicycle, nonmotorized vehicle, truck or lorry, other, skid or rollover, fall form moving vehicle, stationary or fixed object, and other)
- Mobile phone use (yes or no)
- Safety equipment (helmet, seatbelt, and other) used (yes or no)
- Suspected alcohol use with 6 hours of RTI (yes or no)
- Suspected substance use within 6 hours of RTI (yes or no)

**Hospital variables**

- Physiological Assessment
  Glasgow Coma Scale (mild 13-15, moderate 9-12, and severe 3-8)
  Systolic blood pressure (mm Hg; <90, 90-120, and >120)
  Diastolic blood pressure (mm Hg; <60, 60-80, and >80)
  Pulse rate (<60, 60-90, and >90)
  Respiratory rate (<12, 1-18, and >18)
  Lost consciousness (yes or no)
- Patient care
  Emergency room patient (yes or no)
  Anatomy (anatomical region, site of most severe injury; head and neck, face; chest, abdomen, extremities, and external)
  Number of severely injured sites (1-3)
  Pathology (none, concussion, muscle injury, etc)
  Treatment (none, operation, observation, antibiotics, etc)
  Operation (internal-fixation, external-fixation, laparotomy, amputation, etc)
  Intensive care unit (ICU; yes or no)
  Number of days in ICU
- Disposition
  Disposition (died in hospital, discharged to rehabilitation, discharged to home, transferred to another hospital, absconded or left against medical advice, other, and unknown)
- Cost or payment
  Pay fee upon arrival (yes or no)
  Amount paid (local currency)
  Type of service paid for (registration, medicine, x-rays, laboratory tests, blood transfusion, and other)
  Hospitalization cost (local currency)
  Means of payment (had money of his or her own, borrowed from family or friends, took a loan, sold assets, and other)
  Annual household income (local currency)
- Disability history
  Preexisting disability (yes or no)
  Severity of disability or impairment (none, mild, moderate, severe, and extreme)
  Life domains affected (understanding and communicating, getting around, self-care, managing domestic life, getting along with people, engaging in major life areas, and participation in society)

#### Follow-up Data Collection

Using participant contact information provided at the hospital, data collectors called each study participant 1 month, 3 months, and 6 months after hospital discharge to conduct follow-up interviews. Data collectors asked questions about their level of functioning using the WHODAS 2.0 short form (module A). The WHODAS 2.0, a valid and reliable instrument for several patient populations, was developed to allow for comparison across cultures based on a study spanning 19 countries [27]. The instrument consists of 36 items that ask about difficulty in performing activities in the last 30 days, scored on a 5-point Likert scale, as follows: 0=No difficulty, 1=Mild difficulty, 2=Moderate difficulty, 3=Severe difficulty, and 4=Extreme difficulty or inability to do.

WHODAS 2.0’s shortened form consists of 12 items. The short form has been found to be valid and reliable among populations with RTIs in LMICs [29,30]. Scores are calculated for each subscale by adding points from each response. The minimum and maximum total scores of WHODAS 2.0 are 0 to 48, with higher scores indicating higher levels of disability [27]. Three additional questions ask about the number of days participants experienced difficulties in the past 30 days (Table 3).

Module B contains 5 questions. Two questions ask about whether the participants have returned to their preinjury activity and if they have returned to work for those previously employed. Three questions ask about the current use of assistive devices or modifications to their home, the type of equipment or modifications used, and for those reporting not using any, whether they need such assistive devices or modifications. Module C uses 12 selected questions from the CHIEF instrument [28]. This instrument has been implemented and validated in several LMICs [31-36]. The first 3 questions ask whether the participants have the same opportunities as other people to participate and take advantage of the areas of education, employment, and leisure. Nine questions ask about the barriers to participation in activities since the injury. Participants report the frequency of experiencing these barriers (ie, daily, weekly, monthly, monthly, or never). If the item occurs, participants are asked to report the magnitude of this problem (ie, big problem or small problem). The CHIEF questions used in module C were slightly modified from their original form by asking the participants to think about barriers since the occurrence of the injury rather than in the past 12 months.

**Table 3:** List of variables for the follow-up instrument.

Module and measures		Variables
**A^a^**		
	Understanding and communicating (cognition)	Difficulty in concentrating on doing something for 10 minutes Difficulty in learning a new task
	Getting around (mobility)	Difficulty standing for long periods such as 30 min Difficulty in walking a long distance such as a kilometer
	Life activities	Difficulty in taking care of household responsibilities Difficulty in day-to-day work or school
	Participation in society	Difficulty in joining in community activities How emotionally affected by health problems (response options for this item are 0=Not Affected, 1=Mildly Affected, 2=Moderately Affected, 3=Severely Affected, and 4=Extremely Affected)
	Self-care	Difficulty in washing whole body Difficulty in getting dressed
	Getting along with people	Difficulty in dealing with people you do not know Difficulty in maintaining a friendship
	Number of days with difficulties	Overall, in the past 30 days, how many days were these difficulties present? In the past 30 days, for how many days were you totally unable to carry out your usual activities or work because of any health condition? In the past 30 days, not counting the days that you were totally unable, for how many days did you cut back or reduce your usual activities or work because of any health condition?
**B^b^**		
	Return to usual activity	Return to normal life or usual activity (yes or no) Return to work (yes or no)
	Assistive devices	Currently using personal equipment, special adapted devices, and or physical modifications to home (yes or no) Type of equipment used (mobility aids, hearing aids, visual aids, communication aids, cognitive aids, physical modifications, devices for performing tasks, other) Need for assistive devices (yes or no)
**C^c^**		
	Having equal opportunities as other people	Education (yes or no) Employment (yes or no) Recreation or leisure (yes or no)
	Services and assistance barriers^d^	Experience problem with availability of transportation Experience problem with availability of information Experience problem with availability of health care services Need for help at home and not getting it easily
	Attitudes or support barriers^d^	Experience prejudice and discrimination
	Work or school barriers^d^	Experience problem with people’s attitudes at school or work Need help at school or work and did not get it easily
	Policy barriers^d^	Experience difficulty due to government programs and policies
	Physical environment barriers^d^	Experience difficulty with natural environment (temperature, terrain, climate, etc)

^a^Variables from World Health Organization Disability Assessment Survey 2.0 short form (module A). Response options: 0=No Difficulty, 1=Mild Difficulty, 2=Moderate Difficulty, 3=Severe Difficulty, 4=Extreme Difficulty or Cannot Do.

^b^Variables related to return to usual activities and assistive devices (module B).

^c^Variables from Craig Hospital Inventory of Environmental Factors (module C).

^d^Response options: 4=Daily, 3=Weekly, 2=Monthly, 1=Less than Monthly, 0=Never, Not applicable.

#### Data Collection Training, Storage, and Management

Data collectors were trained on the study protocols, and each local team of data collectors and data managers will conduct regular reviews of the data to identify any issues in the data collection process or data quality. The Survey Solutions software developed by the World Bank was used for data collection [37]. Survey Solutions can operate in mixed mode, and data can be collected offline on tablets (computer-assisted personal interviewing), on the web using a web interface (computer-assisted web interviewing), or via phone interviews (computer-assisted web interviewing). Data from the hospital surveillance instrument will be linked to the follow-up data through a unique participant ID. Android-based devices are used to collect, store, and transfer hospital-based and follow-up data. The collected data are transmitted to the survey headquarters for real-time quality control and analysis. The software works offline in areas with limited internet access and later synchronizes data once the internet access is available.

### Ethics Approval

Ethics approval was obtained from the ERES Converge institutional review board in Zambia (reference number 2021-Jan-003), National Ethics Committee for Health Research in Cambodia (reference number 018 NECHR), the institutional review board of the Ethiopian Public Health Association (reference number OG/039/21), the Centre for Injury Prevention and Research in Bangladesh Ethical Review Committee (reference number 2021/01), and the Instituto Nacional de Salud Pública in Mexico (reference number 1729), as well as the George Washington University’s Office of Human Research.

Potential participants were informed of the aims and content of the study and were required to give written, informed consent before enrollment. The participants did not receive any compensation for their participation in the study. All data collected in the Survey Solutions software for this study was deidentified.

### Statistical Analysis

#### Overview

Stata 14 (StataCorp) will be used for the data analysis [38]. First, descriptive statistics will be generated for several variables captured in the hospital-based tool to examine the demographic, clinical, and treatment characteristics of the initial study sample. Descriptive statistics of disability-related variables collected through the follow-up survey will also be generated at each follow-up measurement occasion. Comparing the descriptive statistics of the sample at each time point will help to identify selection bias because of loss to follow-up.

Analyses of the follow-up data will include cross-sectional and linear mixed models to assess the relationship between different predictors (eg, hospital variables) and the study’s 2 key outcome variables (ie, disability score and perceived return to usual activities) at the 3 follow-up time points. The analyses will adjust for differences at baseline (eg, disability history) and confounding variables.

#### Cross-sectional Analysis

To identify significant predictors of disability, a bivariate analysis will be performed at each follow-up time point by regressing the overall disability score against each of the explanatory variables from the hospital-based tool (ie, sociodemographic, preexisting disability, crash circumstances, prehospital, and hospital variables); use of assistive devices from the follow-up tool; and environmental barriers and previous disability score (for the second and third follow-up). Given the large number of variables in the hospital-based tool, there are many potential covariates. A critical assessment will be done to identify the most important independent variables to include in a linear regression model. Variables that show a significant association with the outcome in the bivariate analysis as well as those with associations found in the literature will be retained in the regression model. In addition, testing for multicollinearity in each of the regression models will be performed by determining the variance inflation factor, in which values >10 will be investigated. Collinear variables will be dropped iteratively until the model no longer has variables with a variance inflation factor >10. These measures will ensure that the regression model estimates of the coefficients are stable. This analysis will be repeated using logistic regression and *return to preinjury activity* as the outcome variable.

#### Attrition Analysis

Because disability-related data will be collected longitudinally (repeated measures), it is expected that the sample size at the third follow-up will be smaller than that from the previous follow-ups. An attrition analysis will be performed to test for correlation between demographic and clinical variables and loss to follow-up at each measurement occasion. It is expected that loss to follow-up will be between 4% and 28% at the 6-month follow-up [22,39]. The relationship between nonparticipation in the follow-up and sociodemographic, crash, and clinical variables (collected at the hospital) will be examined to assess whether loss to follow-up from each measurement occasion is associated with the possible predictors of the study outcomes.

#### Longitudinal Analysis Using Mixed Effects Model

Using panel data, the change in the main outcomes of interest (disability score and perceived return to preinjury activity) across the 3 follow-up points will be examined (Table 4). First, attrition analysis will be performed with panel data (all rounds of data collection) to assess whether clinical characteristics such as injury severity and disability score predict loss to follow-up. The loss to follow-up variable will be coded according to the follow-up responses and regressed against injury and disability severity variables. Imputation techniques will also be considered to handle missing data and increase the sample size, depending on the results from the attrition analysis.

**Table 4:** Key outcome variables from the follow-up instrument.

Indicator	Survey questions	Specifications
Disability score	WHODAS^a^ 2.0’s shortened form consists mainly of 12 questions capturing the following domains: understanding and communicating; getting around; self-care; getting along with people; life activities; and participation in society. Response options: 0=No Difficulty, 1=Mild Difficulty, 2=Moderate Difficulty, 3=Severe Difficulty, 4=Extreme Difficulty or Cannot Do.	Scores are calculated for each subscale by adding the points from each response. The minimum and maximum total scores are 0-48, where higher scores indicate higher levels of disability.
Perceived return to preinjury activity or “normal life”	As of today, do you consider yourself to have returned to your normal life or to doing your usual activities your injury? Response options: Yes; No.	This question is intended to capture whether the participant has returned to their usual activities as they see it. Participants may consider themselves to have returned to usual activities or normal life *because of or despite* using an assistive device, whereas other participants may consider themselves not back to “normal” life if they have to use a device or rely on a modification to their physical space. Because this tool is focused on activities and participation, we define “return to normal life” as being able to do the things one was able to do before their injury.

^a^WHODAS: World Health Organization Disability Assessment Survey.

Next, descriptive analysis will be conducted for the complete cases (participants in all 3 follow-up rounds), followed by longitudinal analysis, which will account for the correlation between repeated measures and heterogeneity of variance inherent to longitudinal data. A mixed effects model will be used for the analysis, which accounts for both fixed effects (population-averaged response) and random effects (subject-specific) [40]. This model allows for the comparison of changes in disability scores over time and can accommodate continuous covariates as well as incomplete data [40].

Finally, although we assume a linear relationship in the response over time, this may be relaxed to include a quadratic term for time in the model or a log transformation. *P* values will be considered significant at .1. The longitudinal analysis will be replicated for repeated binary measurements of the return to preinjury activities outcome variable, using logistic regression to model the binary outcome.

## Results

Enrollment in the study began in June 2021 and was completed in April 2022. Follow-up data collection ended in September 2022. Data analysis is currently underway, with results expected for publication in mid-2023.

## Discussion

### Principal Findings

The proposed study will provide an estimate of the burden on patients due to RTI-induced disability in several LMICs. It will also provide a thorough understanding of the rate and severity of RTI-induced disability and its impact on road traffic crash survivors by examining disability scores and perceived return to preinjury activities. The data will identify disparities in recovery from RTIs and return to preinjury activity or “normal life” based on differences in environmental factors surrounding crash survivors after hospitalization. This will provide an opportunity to uncover barriers that impede recovery and return to usual activities, including service and assistance barriers as well as physical, social, and policy barriers.

### Strengths

This study uses a prospective design to develop estimates of disability among patients with RTIs using a validated instrument 1 month, 3 months, and 6 months after injury from 9 hospitals in 5 LMICs. Implementing a multicountry design provides a large sample size that will strengthen the accuracy of our findings, reduce the margin of error, and increase the generalizability of the findings. This prospective design will allow for a better understanding of the predictors that influence the recovery process and provide more efficient estimates of the effects of time-varying covariates, such as injury severity or treatment.

### Limitations

This study has a few limitations. The first limitation is related to sampling. With only a small number of hospitals in each country, our sample is not nationally representative. The study populations that were recruited at these hospitals are not necessarily representative of the injuries and treatment-related factors in each country because of differences in the regional and facility-specific characteristics of the participating hospitals and their patient populations. Thus, the study’s findings will not be generalizable at the country level. In addition, our sample size calculation may not properly reflect the complexity of sample variations, meaning that a sample size of 400 is insufficient for estimating the national disability burden of road traffic crashes, especially for subgroups. Furthermore, because the sample only includes adults with moderate to severe injury, this study will not estimate the full extent of the burden of RTIs because it excludes injuries of children and minors. Second, the COVID-19 pandemic and related restrictions have caused some hospitals to make changes in their inpatient and outpatient case prioritization procedures that may have affected eligibility and recruitment. For example, patients with RTIs with moderate injury who may have normally been admitted to the inpatient ward may have been discharged within 24 hours and scheduled for outpatient procedures. This would render them ineligible for the study and could have created difficulties to achieve the desired sample size as well as potentially cause sampling bias. Even outside of COVID-19, some eligible cases in sampling may have been missed because of premature discharge or transfer of hospital. For example, it is possible that some patients were discharged within 24 hours of admission for unknown reasons despite having moderate to severe RTIs. Similarly, patients who bypassed the emergency department may have also been missed despite our best efforts to recruit eligible patients. Third, a significant portion of the study is longitudinal, which uses phone-based interviews to conduct follow-up data collection, so there is a threat of loss to follow-up. Potential loss to follow-up will reduce the sample size and potentially bias results if missingness is directly linked to health and demographic variables. Fourth, it is likely that the study will have missing data at the hospital level because much of the patient information will be extracted from medical records by nonclinical data collectors. A fifth limitation of this study is that we were not able to collect injury severity score, an important measure of severe injury, because physicians in the participating sites in this study do not routinely calculate this measure. However, other variables were collected, which can act as proxy measures to assess injury severity. Finally, we did not measure the quality of care provided or factors such as psychosocial stress and pain that may directly affect disability-related outcomes.

### Conclusions

This research will be key to expanding the evidence base needed to develop strategies to improve the quality of life and lessen the social and economic burden on those with crash-induced disability. Thus, the findings of this study will help advocate not only for improved road safety but also for informed decision-making by the government to adopt and implement appropriate policies and interventions to address factors contributing to the severity of RTIs and associated disabilities and the contexts under which they occur.

### Dissemination Plan

This research is intended for public health professionals, researchers, trauma care providers, rehabilitation providers, policy makers, and advocates interested in the link between RTIs and disability and rehabilitation. Findings from this study will be reported in peer-reviewed journal articles and conference presentations and shared with the study participants. Publications will be authored jointly by country teams.

## Abbreviations

CHIEF: Craig Hospital Inventory of Environmental Factors
DALY: disability adjusted life year
LMICs: low- and middle-income countries
RTI: road traffic injury
WHODAS 2.0: World Health Organization Disability Assessment Survey 2.0

## Data Availability

The data sets generated and analyzed during this study are available from the corresponding author upon reasonable request.
